# Limitations of the Washington Group Short Set in capturing moderate and severe mobility disabilities

**DOI:** 10.1093/haschl/qxaf015

**Published:** 2025-02-13

**Authors:** Kelsey S Goddard, Jean P Hall

**Affiliations:** University of Kansas, Institute for Health and Disability Policy Studies (KU-IHDPS), 1000 Sunnyside Ave., Room 1052, Lawrence, KS 66045, United States; University of Kansas, Institute for Health and Disability Policy Studies (KU-IHDPS), 1000 Sunnyside Ave., Room 1052, Lawrence, KS 66045, United States

**Keywords:** disability measurement, mobility disability, Washington Group Short Set, health policy, survey methods

## Introduction

The Washington Group Short Set (WG-SS) has become a widely used tool for identifying people with disabilities in federal surveys, aiming to capture people with functional limitations across domains such as vision, hearing, and mobility. However, multiple studies,^1,2^ including our own,^3^ have documented significant limitations in the WG-SS's ability to accurately identify individuals with a variety of disabilities. One rationale for using the WG-SS questions is that they are better at discerning people with more severe disabilities.^4^ However, recent findings^5^ demonstrate that the WG-SS fails to capture many people with severe vision and hearing disabilities (ie, blindness and deafness). While previous analyses have broadly examined the WG-SS's ability to identify people with mobility disabilities,^3^ this paper focuses specifically on those with moderate and severe mobility limitations, particularly individuals who rely on mobility aids. As noted by the National Center for Health Statistics,^6^ “If someone in a wheelchair says they have no difficulty walking, then something is not right [with the WG-SS questions].” The current study reveals that the WG-SS misses a substantial portion of this group, raising critical concerns about the validity evidence of the WG-SS as a comprehensive measure for capturing disability prevalence and severity.

## Methods

**Figure 1.**
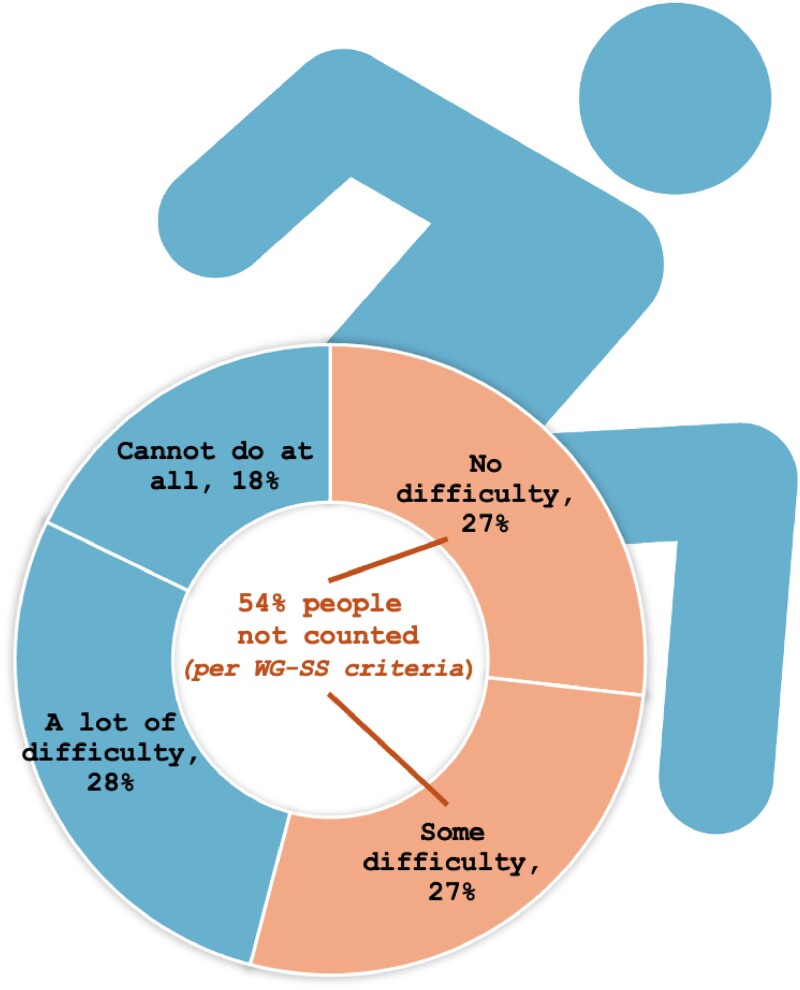
Distribution of responses to the WG-SS mobility question, “Do you have difficulty walking or climbing steps?” among NHIS respondents who reported “using any equipment or receiving help with walking, climbing steps, or moving around” (Sample Adult File, 2010-2018; *N* = 12 851). Responses are divided into 4 categories: “No difficulty” (27%), “Some difficulty” (27%), “A lot of difficulty” (28%), and “Cannot do at all” (18%). According to WG-SS criteria, 54% of this group is categorized as nondisabled, as they reported “no difficulty” or “some difficulty.”

**Figure 2.**
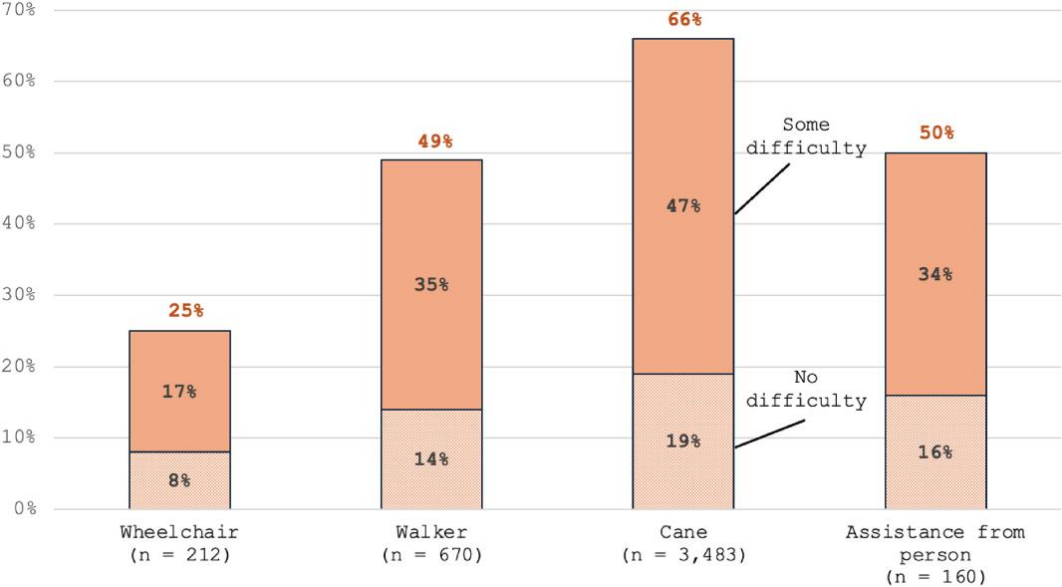
Percentage of people who reported using a single type of mobility aid—either a wheelchair (*N* = 212), walker (*N* = 670), cane (*N* = 3483), or assistance from another person (*N* = 160)—and their responses to the WG-SS mobility question, “Do you have difficulty walking or climbing steps?” (NHIS Sample Adult File, 2010-2018). Each bar shows the distribution of responses classified as “No difficulty” or “Some difficulty” (shaded), which, according to WG-SS criteria, categorizes them as nondisabled despite reported use of mobility aids. The proportions indicate the percentage of respondents within each group who are effectively not counted as having a mobility-related disability by the WG-SS.

We used publicly available data from the National Health Interview Survey (NHIS) 2010-2018 Sample Adult Files, accessed through IPUMS,^7^ to examine the WG-SS's performance in identifying people with mobility-related disabilities. This period aligns with prior analyses evaluating the WG-SS question set.^5^ To capture a range of mobility support needs, we analyzed 2 distinct respondent groups. The first group included those who reported “using any equipment or receiving help with walking, climbing steps, or moving around” (Figure 1, *N* = 12 851). The second group included individuals who reported using only 1 type of mobility aid—either a wheelchair (*n* = 212), walker (*n* = 670), cane (*n* = 3483), or assistance from another person (*n* = 160) (Figure 2). To ensure these samples were independent, we excluded individuals who reported using multiple types of mobility aids (eg, both a wheelchair and a walker). We then analyzed their responses to the WG-SS mobility question, “Do you have difficulty walking or climbing steps?”, which serves as the WG-SS's single item for capturing mobility limitations. According to WG-SS criteria, respondents are categorized as nondisabled if they answer “no difficulty” or “some difficulty” to this question.^8^ Using descriptive statistics, we calculated the proportion in each group categorized as nondisabled despite their reported use of mobility aids.

## Results

Our NHIS data analysis reveals substantial limitations in the WG-SS's ability to identify individuals with moderate and severe mobility-related disabilities. Among those who reported using any equipment or receiving help with walking, climbing steps, or moving around, 54% indicated “no difficulty” or “some difficulty” on the WG-SS mobility question. Specifically, 25% of wheelchair users, 49% of walker users, 66% of cane users, and 50% of those needing assistance from a person would not be counted as disabled according to the WG-SS. These results suggest that the WG-SS may systematically misclassify a significant portion of individuals with substantial mobility impairments, underscoring its limitations in accurately capturing the prevalence and severity of mobility-related disabilities.

## Discussion

The undercounting of individuals with severe disabilities by the WG-SS has considerable policy implications. Federal disability data, often derived from WG-SS-based surveys, inform funding decisions for programs and services. When people with disabilities—whether they have more severe disabilities, as implied by wheelchair use, or less severe ones, as implied by cane use—are systematically underrepresented, resources may be insufficiently allocated.^9^ This lack of resources limits access to essential services and equitable social and health outcomes. Furthermore, excluding people across the spectrum of mobility-related disabilities skews our understanding of health disparities within this population, potentially distorting views of their experiences and needs.

Mobility-related disabilities affect many aspects of life, including employment,^10^ access to physical environments,^11^ and social participation,^12^ and they often worsen over time.^13,14^ By failing to count individuals with both severe and moderate mobility limitations, the WG-SS contributes to an incomplete picture of disability prevalence and severity. This gap is concerning because individuals who might initially require minimal support may experience increasing limitations over time and could benefit from earlier interventions and supports.^15^

While this paper addresses only mobility disabilities, other research has demonstrated that a single, broad disability question captures more people with *all* types of disabilities, including mobility, cognitive, intellectual, and psychiatric disabilities, than does the WG-SS.^3,16^ In addition, follow-up disability self-categorization or write-in questions provide more specificity on disability type than the WG-SS.

These findings, which build on prior research, underscore the need for immediate revisions in how disability is measured in federal surveys such as the NHIS. Policymakers should consider more inclusive approaches that consistently capture individuals with disabilities across a range of severity levels.^3,16,17^ Given the cumulative evidence of WG-SS undercounts across chronic illness, psychiatric, sensory, and mobility disabilities, it is clear that the WG-SS is insufficient for capturing the true prevalence and needs of people with disabilities.

## Supplementary Material

qxaf015_Supplementary_Data
